# An Analysis of the Optimal Mix of Global Energy Resources and the Potential Need for Geoengineering Using the CEAGOM Model

**DOI:** 10.1002/gch2.201700040

**Published:** 2017-11-13

**Authors:** John G. Anasis, Mohammad Aslam Khan Khalil, George G. Lendaris, Christopher L. Butenhoff, Randall Bluffstone

**Affiliations:** ^1^ Systems Science Program College of Liberal Arts & Sciences Portland State University PO Box 751 Portland OR 97207 USA; ^2^ Physics Department College of Liberal Arts & Sciences Portland State University PO Box 751 Portland OR 97207 USA; ^3^ Economics Department College of Urban and Public Affairs Portland State University PO Box 751 Portland OR 97207 USA

**Keywords:** climate targets, energy resource optimization, geoengineering

## Abstract

Humanity faces tremendous challenges as a result of anthropogenic climate change caused by greenhouse gas emissions. The mix of resources deployed in order to meet the energy needs of a growing global population is key to addressing the climate change issue. The goal of this research is to examine the optimal mix of energy resources that should be deployed to meet a forecast global energy demand while still meeting desired climate targets. The research includes the unique feature of examining the role that geoengineering can play in this optimization. The results show that some form of geoengineering is likely to be needed by the middle of the 21st century as part of the optimal energy strategy in order to meet a specified climate goal of 580 ppm CO_2_‐eq greenhouse gas concentration (or ≈2 °C average global temperature rise). The optimal energy mix would need to rely on energy efficiency, nuclear, geothermal, hydro, and wind energy for over 50% of global energy needs. In addition, the overall cost of the optimal energy mix is sensitive to the assumed amount of achievable energy efficiency, carbon taxes, deployment of electric vehicles, and the assumed discount rate.

## 1 Introduction

One of the greatest challenges that humanity will face over the coming decades is global climate change. As a result of increased greenhouse gas (GHG) emissions, the world is on track to sustain an increase in average global temperature of up to 4 °C by the end of the 21st century.^1^ The implications of this temperature rise are not entirely clear as yet; however, the impacts to much of the world's population and to the environment are almost certain to be widespread and serious.^2^, ^3^ On the other hand, the steps that would be required to reduce greenhouse gas emissions through reductions in the use of fossil fuels would also involve large economic and social impacts.^4^ This imposes significant political inertia and resistance on the part of both governments and the general public to take any substantial action in the near term to cut greenhouse gas emissions. Frequently voiced concerns over efforts to reduce emissions include loss of jobs, increases in energy prices, ceding competitive advantage to economic rivals in other countries, and potential legal risks. All these arguments can be clearly seen in the reaction to the Paris Accord.^5^, ^6^

This serious challenge has led many researchers to suggest that various kinds of artificial measures could be taken that would reduce the radiative forcing effects (defined as the net change in radiation at the troposphere without stratospheric temperature adjustment) caused by the burning of fossil fuels. These various proposals are collectively known as geoengineering. A number of geoengineering proposals have been suggested over the years. Four of the more technically feasible geoengineering options were included in this study and are summarized in **Table** **1**. More specific details on these geoengineering options can be found in the references cited in the table.

**Table 1:** Summary of geoengineering options

Geoengineering option	Description	Advantages	Disadvantages
Sulfur injection^7^, ^8^	Injection of sulfur aerosols into the stratosphere by aircraft	Concept demonstrated by volcanic eruptions; delivery technology in the form of tanker aircraft is proven	Does not reduce the actual atmospheric greenhouse gas concentration, so must be constantly deployed
Sea spray injection^9^, ^10^	Injection of sea water droplets into the air to thicken low‐level maritime clouds, thereby increasing albedo	Enhances an existing natural process; does not introduce any chemicals into the environment	Does not reduce the actual atmospheric greenhouse gas concentration, so must be constantly deployed; unproven delivery technology; geographically limited
Ocean fertilization^11^, ^12^, ^13^, ^14^	Addition of nutrients (such as iron, nitrogen, or phosphorus) to the ocean to enhance the natural biological carbon pump	Enhances an existing natural process; actually removes CO_2_ from the atmosphere; easily deployed using tanker ships	Potential adverse impacts to marine environments
Tree planting	Planting trees to absorb CO_2_	No special technology required; additional economic and environmental benefits, such as lumber and erosion protection	Net CO_2_ absorption stops once trees mature; significant land area required which could compete with other uses

The concept of geoengineering is controversial. There are a number of potential risks associated with the various proposals, especially those (such as sulfur or sea spray injection) which reduce radiative forcing without reducing CO_2_ concentrations. These risks include possible major changes in precipitation patterns and continued increased acidification of the world's oceans.^15^, ^16^, ^17^, ^18^, ^19^

Despite the controversy surrounding geoengineering, there is a strong chance that at least some degree of geoengineering may have to be deployed at some point once the effects of increased GHG concentrations and the associated changes to global climate become more apparent. Some authors have suggested that geoengineering technology be explored for emergency preparedness in the event it becomes clear that the earth's climate is heading for a potentially catastrophic outcome.^20^, ^21^, ^22^, ^23^ It has even been suggested that the positive aspects of geoengineering may be attractive enough for some countries that they would be willing to undertake some geoengineering efforts unilaterally.^24^ For these reasons, the National Academy of Sciences has recommended that careful research into geoengineering and its potential impacts be undertaken.^25^, ^26^

Given the dangers of climate change, the goal of this research was to examine what potential global energy policies might be undertaken to optimally meet anticipated global energy needs while also meeting specified climate targets. The unique feature of this research was the explicit inclusion of potential geoengineering options as part of the optimization. No previous study has included geoengineering options in global energy resource optimization strategies.

The following sections of this paper are structured as follows. An overview of the model used for this study is provided in Section 2. Next, a summary of the key assumptions, scenarios evaluated, and data used is provided. Finally, the results of the simulations are discussed, as well as the corresponding conclusions drawn from them.

## 2 Methodology

### 2.1 Model Description

In order to perform the desired optimization analysis, a new model was developed called the combined energy and geoengineering optimization model (CEAGOM). There were several motivations behind the development of the CEAGOM model for this analysis. First of all, existing integrated assessment models (e.g., the integrated MARKAL‐EFOM system (TIMES), global change assessment model (GCAM), and national energy modeling system (NEMS)) did not readily accommodate the range of geoengineering options that were desired to be included as part of the research.^27^, ^28^, ^29^ In addition, the analysis required a model that was computationally inexpensive so as to allow extensive sensitivity studies. For example, the cases run on CEAGOM for this study ran in less than 5 min on a standard Intel i3 laptop computer. Similar scenarios run employing the widely used TIMES model had reported run times between 80 and 440 min (computer type unspecified by the authors).^30^ Finally, CEAGOM was developed using the readily available commercial MATLAB software. The resulting model was applicable not only to a global analysis, it could also be used to perform a regional analysis, such as an examination of the 2014 emissions deal between the U.S. and China. It is important to note that CEAGOM is not a forecasting model. It is an optimization model designed to determine the set of energy resources and geoengineering that should be deployed in order to meet a given energy forecast at the lowest cost subject to specified resource and climate constraints. By contrast, integrated assessment models (IAMs), such as GCAM, do not optimize resources to minimize costs. Rather, they reach a resource solution by “clearing the markets,” i.e., iterating energy prices until supply meets demand based on a set of supply–demand curves for the various resources and end uses incorporated in the model.

**Figure 1:**
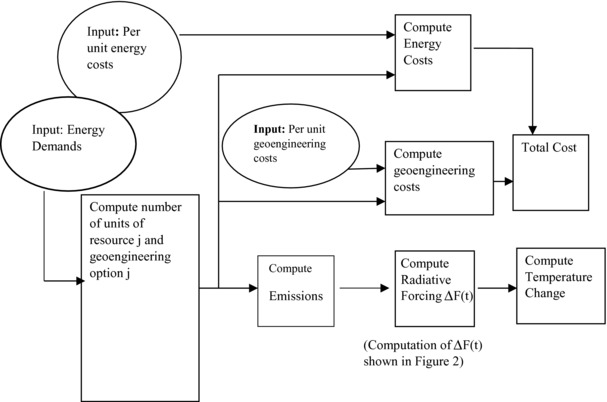
Schematic of CEAGOM components and data flows.

**Figure 2:**
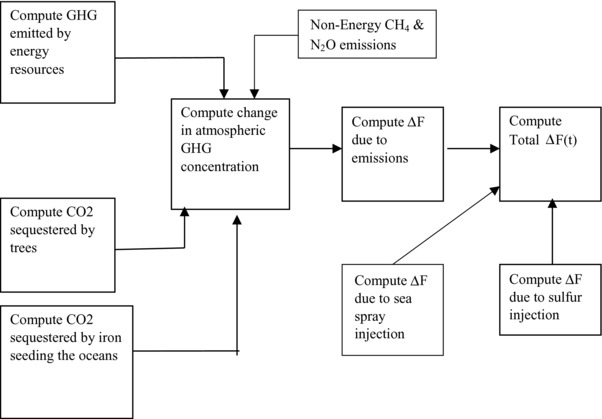
Schematic of CEAGOM radiative forcing computation.

**Figures** **1** and **2** describe the basic structure of the CEAGOM model. CEAGOM consists for four sub‐models. The first is an energy model that incorporates all the key parameters for each energy and geoengineering resource. These include per unit costs, energy conversion efficiencies, per unit emissions, resource lifetimes, total resource availability, and maximum allowable annual resource increase or decrease. The energy model also includes the energy demand forecast to be met. The second sub‐model is a climate model. This component computes emissions and the associated changes in greenhouse gas concentration, radiative forcing, and global average temperature change. The third sub‐model is the economics model. This component computes the cost of the energy and geoengineering resources that are deployed to meet the energy demand. These costs are computed on both a net present value and nominal cost basis. The final component of CEAGOM is the engine which performs the actual optimization calculation. The optimization engine used by CEAGOM is the thoroughly tested fmincon constrained nonlinear optimization interior point subroutine developed by The Mathworks, Inc. The optimization engine uses both resource availability and a climate limit as constraints. CEAGOM allows the user to specify total emissions, greenhouse gas concentration, or average global temperature change as the climate constraint. A full description of the CEAGOM model, including source code and user guide, is available through the Portland State University Library.^31^

The analysis incorporated an assumed set of proven energy resources that would be available in order to meet the specified energy demand. The analysis only used proven energy resources. These resources were oil, coal, natural gas, nuclear power, hydroelectric, solar photovoltaic (solar‐PV), solar thermal (also known as concentrated solar power), geothermal, wind, biomass, biofuel, and energy efficiency. This was done because their operating characteristics and costs are known. Other proposed technologies, such as carbon capture and storage, were not included in the analysis. This was because their viability has not been fully shown yet, and their performance and costs are not well understood.^32^ In addition, the known availability of each assumed energy resource was included in the simulations as a constraint along with the climate limit. Four geoengineering options were incorporated in the analysis. They were sulfur injection, sea spray injection, iron seeding, and tree planting.

### 2.2 Scenarios

#### 2.2.1 Scenario Overview

The scenarios examined in this analysis are described in **Table** **2**. A baseline analysis was performed by optimizing energy resources assuming no climate limit (signified by the NL suffix). This provided a baseline energy solution based strictly on economics and resource availability. The case was then rerun with the desired climate limit constraint (signified by the CL suffix). The process was repeated for the full range of sensitivity scenarios shown in Table 2. The scenario names are short abbreviations that indicate the parameter or condition that was varied for that particular sensitivity case.

**Table 2:** Scenario descriptions

Scenarios	Description
Base‐NL	Baseline cases
Base‐CL	
1%‐NL	1% discount rate
1%‐CL	
10%‐NL	10% discount rate
10%‐CL	
2bha‐NL	Only 2 billion hectares available for biomass, biofuel, and tree planting
2bha‐CL	
10mha‐CL	10 million hectares of trees planted annually between 2025 and 2075
500mha‐CL	500 million hectares of trees assumed planted in 2050
Eff‐NL	Less achievable energy efficiency—10% for first 50 years and 15% for the second 50 years
Eff‐CL	
Elcar‐NL	Increased use of electric cars—reduce liquid energy demand by 0.25% per year to 2080 (20% reduction) and hold for remaining 20 years. Corresponding increase in electric energy demand
Elcar‐CL	
GeoPen‐CL	$250 billion penalty applied to each unit of sulfur injection, sea spray injection, or ocean fertilization deployed
GeoPen2‐CL	$1 trillion per ton penalty on each ton of sulfur injection deployed. $250 billion penalty applied to each unit of sea spray or ocean fertilization deployed
NoGeo	Case to find the lowest CO_2_‐eq concentration achievable with no geoengineering
NoGeo2	Case to find the lowest CO_2_‐eq concentration achievable with no geoengineering except for tree planting
NoNuke‐NL	No allowed increase in the amount of nuclear power deployed. Existing plants could still be deployed and replaced in kind at the end of their service lives
NoNuke‐CL	
NukePen‐NL	$26.8 billion TW^−1^ penalty cost placed on nuclear power
NukePen‐CL	
Tax‐NL	$100 per ton carbon tax on oil, coal, and natural gas
Tax‐CL	
Tax50‐NL	$50 per ton carbon tax on oil, coal, and natural gas
Tax50‐CL	

#### 2.2.2 Climate Limit

The climate limit chosen was based on the representative concentration pathways (RCP) adopted by the Intergovernmental Panel on Climate Change (IPCC).^33^, ^34^ The RCPs are a series of storylines that describe climate limits in terms of ranges of both greenhouse gas concentrations and cumulative emissions. The concentration limits essentially become limits on the resulting radiative forcing which, in turn, becomes a limit on overall global temperature rise. For purposes of this research, the lowest concentration value of 580 ppm from RCP4.5 was chosen as the benchmark limit to align with the 2 °C reduction target of the Paris climate agreement. This concentration corresponds to a radiative forcing of 3.93 W m^−2^ and an associated global average temperature rise of 1.97 °C relative to preindustrial levels.^35^ The IPCC does note that the actual global average temperature rise for any given greenhouse has concentration could vary considerably depending on carbon cycle and climate system uncertainties. As described in refs. 33 and 34, the global temperature rise could be between 1.4 and 3.6 °C for a 580 ppm CO_2_‐eq concentration. Hence, this research used concentration rather than temperature rise as the climate limit.

#### 2.2.3 Discount Rate

A discount rate of 4% was assumed for the majority of the scenarios. From 1990 to 2016, the 12 month London Interbank Offer Rate has ranged from over 8% to slightly under 1%.^36^ Similarly, the US Prime Rate has ranged from 10 to 3.25% over the same time period.^37^ The 4% value was chosen as a reasonable mid‐range value.

Sensitivity runs were also made assuming discount rates of 1 and 10% (cases 1%In‐NL, 1%In‐CL, 10%In‐NL, and 10%In‐CL) since interest rates can fluctuate considerably over time. The discount rate is important, because it represents an estimate of a society's willingness to trade off present benefits for future gains. It is therefore a fundamental economic behavioral feature. Furthermore, with timeframes beyond about 30 years, the choice of discount rate can greatly affect estimates. It is, therefore, not merely a technical parameter, but the conversion rate between the present and the future with dramatic consequences for the economic analysis.

#### 2.2.4 Energy Efficiency

In addition, the majority of the scenarios assumed that energy efficiency could meet up to 20% of annual energy demand for the first 50 years of the simulation and up to 25% of annual energy demand for the final 50 years, unless otherwise noted. This assumed increase in efficiency was meant to simulate improvements in technology. However, it is possible that these levels of energy efficiency might be achieved. Sensitivity cases Eff‐NL and Eff‐CL were run to explore the impacts if only 10% of energy demand for the first 50 years and 15% of demand for the second 50 years of the simulation could be achieved.

#### 2.2.5 Available Land for Tree, Biofuel, and Biomass Planting

The scenarios generally assumed that up to 3 billion hectares of land would be available to produce biomass and biofuel, as well as for tree planting if called upon as a geoengineering measure based on estimates of available arable land.^38^ This is a considerable amount of land which could result in competition with other important uses, particularly food production. Hence, sensitivity cases 2bha‐NL and 2bha‐CL were run assuming only 2 billion hectares of land available for biofuel, biomass, and tree planting.

#### 2.2.6 Nuclear Power

The role of nuclear power in the global energy mix has been controversial for many years, especially after the major accidents at Three Mile Island, Chernobyl, and Fukushima. A set of sensitivity cases were run to examine what effect limitations on the use of nuclear power might have on the optimal global energy mix and associated climate impacts. The first of these were cases NoNuke‐NL and NoNuke‐CL. These scenarios assumed that the total amount of nuclear power deployed could not increase above the initial level in the scenario, but did allow existing nuclear resources to be replaced once they reached the end of their lifetimes. Cases NukePen‐NL and NukePen‐CL, on the other hand, did not restrict the total amount of nuclear resources that could be deployed, but did place a penalty cost of $26.8 billion TW^−1^ of nuclear capacity deployed. This penalty cost was meant to capture the cost of a major nuclear accident (refer to ref. 31 for calculation of this penalty).

#### 2.2.7 Electric Vehicles

There has been considerable discussion over the role electric vehicles could play as a means of mitigating GHG emissions.^39^ Hence, two sensitivity cases, Elcar‐NL and Elcar‐CL, were run to examine the impact that a significant shift to electric vehicles could have on the global resource mix and climate impacts. This was done by assuming that 0.25% of the global demand for liquid fuel was reduced per year for the first 80 years of the simulation, thus reducing liquid fuel demand by 20% in the year 2080. This 20% reduction was then held for the final 20 years of the simulation. A corresponding increase in the demand for electric energy was made over this same time period.

#### 2.2.8 Carbon Taxes

Carbon taxes are another widely discussed topic in debates over climate change mitigation. In order to explore their potential impact, two potential carbon taxes were examined. One was a $50 per ton tax (cases Tax50‐NL and Tax50‐CL) and much more significant $100 per ton tax (cases Tax‐NL and Tax‐CL). These taxes were applied to the emissions from any coal, oil, or natural gas resources deployed in the simulations.

#### 2.2.9 Special Geoengineering Scenarios

A set of sensitivity cases examining certain aspects of geoengineering were included as part of the analysis. One set of sensitivity cases, 10mha‐CL and 500mha‐CL, was designed to examine the potential effect of large scale tree planting on overall emissions and global temperature rise. The 10mha‐CL case assumed that 10 million hectares of trees were planted from 2025 through 2075 for a total of 500 million hectares. Case 500mha‐CL was a more theoretical case that assumes the entire 500 million hectares of trees were all planted in the year 2050 to see if that would have a more significant impact on emissions over the second half of the simulation.

Another set of special geoengineering sensitivity cases were GeoPen‐CL and GeoPen2‐CL. These cases assumed significant penalty costs on the sea spray, sulfur injection, and ocean fertilization options in order to address the potentially significant risks associated with these forms of geoengineering.

The no geoengineering scenarios NoGeo and NoGeo2 were unique cases. These two scenarios were used to determine the lowest CO_2_‐eq concentration limit that could be held without the use of geoengineering (the NoGeo2 scenario did allow the use of tree planting; the NoGeo scenario did not). The purpose was to examine if significant radiative forcing reductions could be achieved by shifting the energy resource mix while still meeting the original energy forecast. These two scenarios assumed faster ramp‐up rates on nonfossil fuel resources (such as nuclear and renewable energy) than the other scenarios in this study. They also assumed an energy efficiency potential of up to 37% of demand based on the work of Krewitt et al.^40^ Both scenarios assumed a $100 per ton carbon tax to discourage fossil fuel use. Scenario NoGeo assumed no geoengineering. Scenario NoGeo2 assumed that 75 million hectares of trees per year were planted over the 40 year period from 2035 to 2075. Up to 4 billion hectares were assumed available for biofuel and biomass production and, in the case of NoGeo2, for tree planting.

## 3 Input Data

The energy forecast data used in this analysis were the publicly available AMPERE2‐Base‐Conv‐OPT scenario of the IMACLIM v1.1 model found in the IPCC's Fifth Assessment Report (AR5) database.^41^ This scenario was chosen because it represented a fairly high energy demand and did not already assume significant changes in technology, thereby providing a conservative case that would serve to clearly illustrate the potential challenges facing the world regarding energy and climate change.

Table S.1 in the Supporting Information summarizes all the cost data used for both the energy and geoengineering resources that were part of this study. The table also shows the efficiency and capacity factors assumed for each of the energy resources, as well as the sources of the data. This input data, as well as a detailed description of all study results, are available with the CEAGOM code at the previously cited ref. 31 to the Portland State University Library.

## 4 Results

**Table** **3** summarizes the emissions, radiative forcing, and global average temperature rise for those scenarios where no climate target constraints were imposed. Table 3 also includes the results of the NoGeo and NoGeo2 no geoengineering scenarios. In all cases where geoengineering was deployed in order to meet the specified climate limit, the global temperature rise was held to a maximum value of 1.97 °C (which corresponds to a 580 ppm concentration limit) throughout the course of the simulation.

**Table 3:** Climate results with no climate limits

Scenario	Actual cumulative emissions (Gt CO_2_‐eq)	Radiative forcing in 2100 [W m^−2^]	Temperature rise from preindustrial by 2100 [°C]
NoNuke‐NL	6250	6.34	3.17
Eff‐NL	6029	6.18	3.09
2bha‐NL	5669	6.01	3.00
10%In‐NL	5746	6.00	2.98
NukePen‐NL	5722	5.99	3.00
Base‐NL	5642	5.98	2.99
1%In‐NL	5569	5.96	2.98
Tax‐NL50	5543	5.93	2.97
Elcar‐NL	5524	5.89	2.95
Tax‐NL	5159	5.75	2.88
NoGeo	4193	5.12	2.56
NoGeo2	3900	4.91	2.45

**Figure 3:**
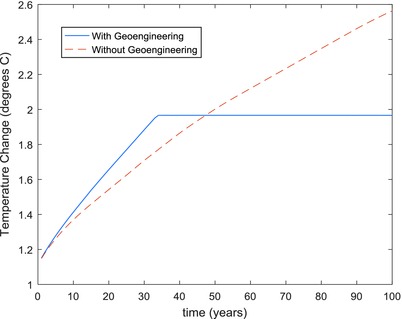
Comparison of temperature rise in baseline case with geoengineering (Base‐CL) and case without geoengineering (NoGeo).

In the NoGeo and NoGeo2 cases which did not include geoengineering, on the other hand, the global temperature could not be stabilized. **Figure** **3** compares the global temperature rise for the NoGeo scenario to the temperature rise for the baseline case with geoengineering. The global average temperature rise reached 2.56 °C by the year 2100. The temperature rise for the NoGeo2 scenario which included tree planting was slightly less reaching 2.45 °C by the year 2100, but basically followed the same trajectory.

**Table** **4** summarizes the amount of geoengineering required in order to meet the 580 ppm CO_2_‐eq concentration limit for the scenarios where that limit was imposed. The amount of sea spray and sulfur injection geoengineering deployed was significant; however, it was still well within the realm of what is theoretically feasible (see refs. 8 and 10). Also of note is that the optimal solution still called for significant amounts of geoengineering even when a substantial penalty was applied as shown by the results for the GeoPen‐CL and GeoPen2‐CL cases.

**Table 4:** Geoengineering required to meet climate limits

Scenario	Geoengineering maximum deployed
Base‐CL	Sea spray
	24.8 m^3^ s^−1^
10mha‐CL	Trees
	10^7^ ha per year for 50 years
	Sea spray
	24.6 m^3^ s^−1^
500mha‐CL	Trees
	500 million ha in 2050
	Sea spray
	24.6 m^3^ s^−1^
GeoPen‐CL	Sulfur
	2.45 million tons
GeoPen2‐CL	Sulfur
	2.45 million tons
1%In‐CL	Sea spray
	24.6 m^3^ s^−1^
10%In‐CL	Sea spray
	25.2 m^3^ s^−1^
2bha‐CL	Sea spray
	25.2 m^3^ s^−1^
Eff‐CL	Sea spray
	27.4 m^3^ s^−1^
Elcar‐CL	Sea spray
	23.8 m^3^ s^−1^
NoNuke‐CL	Sea spray
	29.3 m^3^ s^−1^
NukePen‐CL	Sea spray
	25.0 m^3^ s^−1^
Tax‐CL	Sea spray
	22.1 m^3^ s^−1^
Tax50‐CL	Sea spray
	24.3 m^3^ s^−1^

**Table** **5** shows the corresponding total costs for all the energy and geoengineering resources deployed over each 100 year simulation with the 580 ppm CO_2_‐eq concentration limit, as well as the NoGeo and NoGeo2 scenarios. Both the net present value (NPV) and nominal value of the costs are shown. The NPV and nominal costs of the NL scenarios were practically the same as those for their corresponding CL cases. This was due to the extremely low cost of the geoengineering relative to the overall cost of the energy resources needed to meet the energy demand over the simulation period. This will be discussed further in the next section.

**Table 5:** Scenario NPV and total nominal costs

Scenario	NPV ($ trillion)	Total nominal cost ($ trillion)
Base‐CL	124.02	1264.46
10mha‐CL	136.10	1758.31
500mha‐CL	136.09	1758.33
GeoPen‐CL	125.46	1288.0
GeoPen2‐CL	129.77	1358.49
1%In‐CL	423.05	755.87
10%In‐CL	29.85	1495.32
2bha‐CL	149.64	2259.34
Eff‐CL	185.30	2918.36
Elcar‐CL	109.44	702.71
NoGeo	104.75	555.82
NoGeo2	105.25	556.90
NoNuke‐CL	187.18	3332.22
NukePen‐CL	130.12	1428.37
Tax‐CL	114.93	704.48
Ta50‐CL	115.14	862.68

Interestingly, the electric vehicle sensitivity case showed the lowest overall cost on a net present value basis of all the scenarios which assumed a 4% discount rate. Also of note is the relatively high cost in the scenarios that assumed large scale tree planting (10mha‐CL and 500mha‐CL), as well as the case where only 2 billion hectares of land was available for biofuel/biomass production and tree planting (2bha‐CL).

In addition to the direct energy resource costs, the four scenarios that assumed a global carbon tax showed that the tax would generate considerable revenue on both a net present value and nominal basis as summarized in **Table** **6**.

**Table 6:** Carbon tax revenues

Scenario	NPV ($ trillion)	Total nominal cost ($ trillion)
NoGeo	47.17	228.69
NoGeo2	47.18	229.50
Tax‐CL	66.88	319.93
Tax50‐CL	36.68	176.19

**Figure 4:**
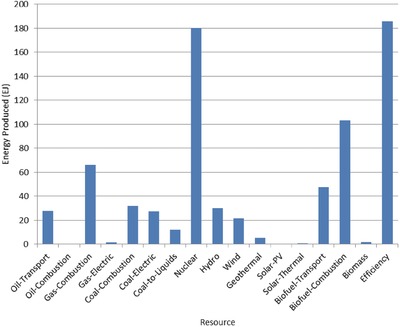
Resource mix in year 2100 for baseline and geoengineering penalty cases (Base‐NL, Base‐CL, GeoPen‐CL, and GeoPen2‐CL).

**Figure 5:**
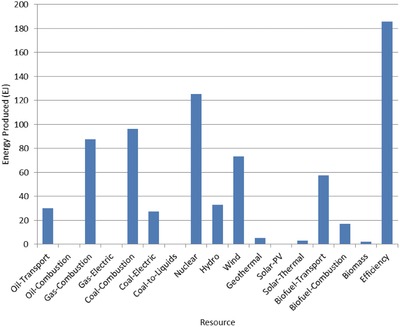
Resource mix in year 2100 for 1% discount rate cases (1%In‐NL and 1%In‐CL).

**Figure 6:**
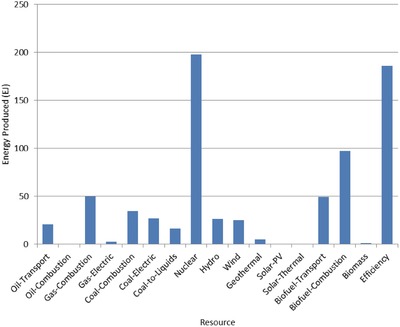
Resource mix in year 2100 for 10% discount rate cases (10%In‐NL and 10%In‐CL).

**Figure 7:**
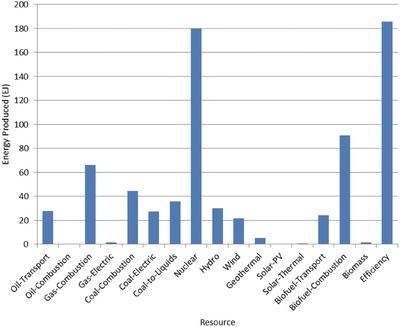
Resource mix in year 2100 for cases with 2 billion hectares available for tree and biofuel/biomass (2bha‐NL and 2bha‐CL).

**Figure 8:**
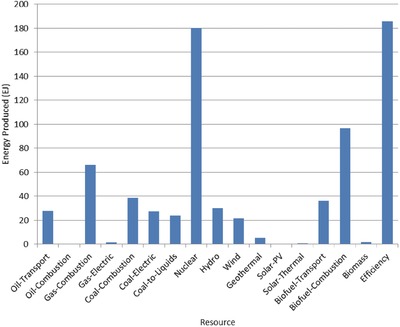
Resource mix in year 2100 for case with 10 million hectares of trees planted annually (10mha‐CL).

**Figure 9:**
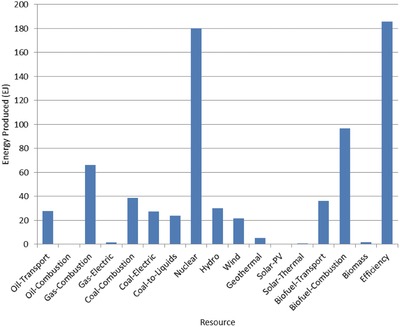
Resource mix in year 2100 for case with 500 million hectares of trees planted in 2050 (500mha‐CL).

**Figure 10:**
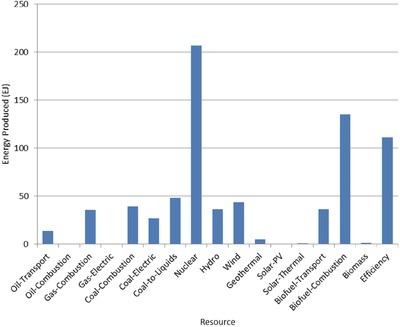
Resource mix in year 2100 for cases with reduced energy efficiency (Eff‐NL and Eff‐CL).

**Figure 11:**
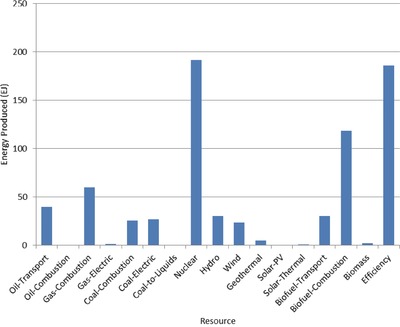
Resource mix in year 2100 for cases with significant electric vehicle usage (Elcar‐NL and Elcar‐CL).

**Figure 12:**
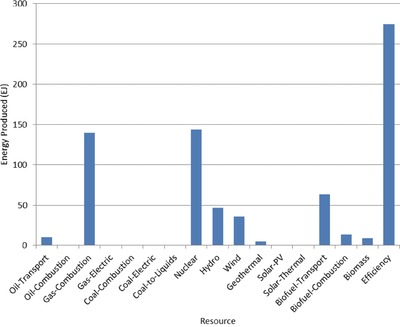
Resource mix in year 2100 for case with no geoengineering (NoGeo).

**Figure 13:**
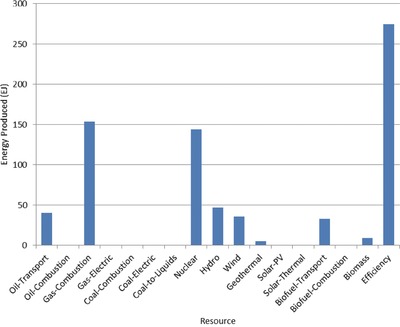
Resource mix in year 2100 for case with no geoengineering except tree planting (NoGeo2).

**Figure 14:**
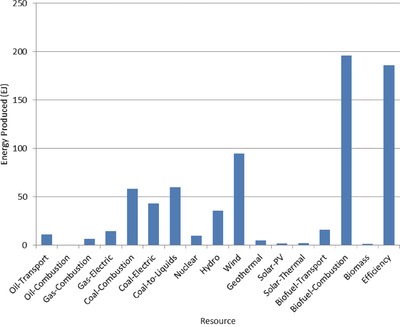
Resource mix in year 2100 for cases with no increase in nuclear power (NoNuke‐NL and NoNuke‐CL).

**Figure 15:**
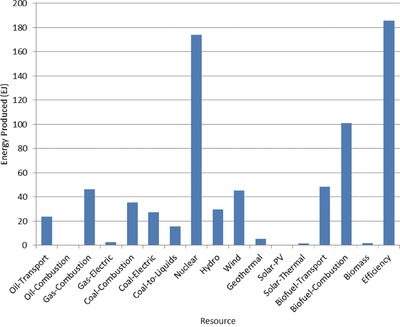
Resource mix in year 2100 for cases with a penalty on nuclear power (NukePen‐NL and NukePen‐CL).

**Figure 16:**
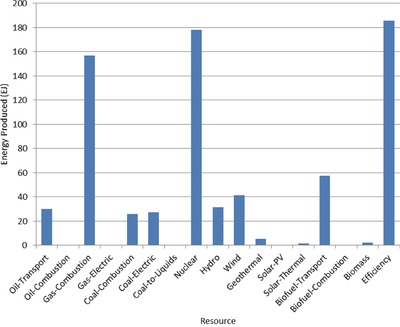
Resource mix in year 2100 for cases with a $100 per ton carbon tax (Tax‐NL and Tax‐CL).

**Figure 17:**
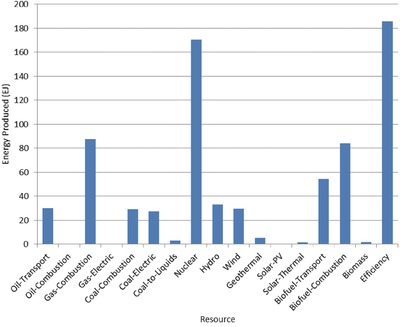
Resource mix in year 2100 for cases with a $50 per ton carbon tax (Tax50‐NL and Tax50‐CL).

The optimal mix of energy resources deployed in the year 2100 for each scenario is summarized in **Figures** **4**–**17**. It should be noted that the energy mix in cases where geoengineering was deployed was the same as the corresponding case with no 580 ppm CO_2_‐eq concentration limit as shown in Figures 4, 5, 6, 7, 10, 11, 14, 15, 16, and 17. This makes sense since it was the geoengineering rather than any change in resource deployments that allowed the 580 ppm CO_2_‐eq concentration limit to be met. The figures show the resource mix in terms of final energy produced from each resource, i.e., after taking resource efficiency into account. This allows the contributions of the resources in meeting the energy demand to be directly compared.

Also of note in these results is how consistently the optimization called for the use of energy efficiency and nuclear power across the range of scenarios examined. Furthermore, as shown by the cost results in Table 5, the highest overall costs on a net present value basis were for those scenarios where the available amount of either nuclear power or energy efficiency was limited.

## 5 Discussion

The most striking result from this analysis was the likely need for some form of geoengineering to be deployed sometime by the middle of this century in order to limit radiative forcing and the associated rise in global temperature. The cases without sulfur injection, sea spray injection, or ocean fertilization (NoGeo and NoGeo2) showed that, even with extremely aggressive and rapid changes in the global mix of energy resources, global temperature rise by the year 2100 would still exceed 2.4 °C. Moreover the radiative forcing and temperature rise were by no means stabilized at that point. They were on a trajectory that was continuing to increase at a substantial rate.

Furthermore, the results from the NoGeo2, 10mha‐CL, and 500mha‐CL analyses also showed that the impact of significant amounts of tree planting on the overall greenhouse gas concentration and resulting global temperature rise may be marginal. The amount of CO_2_ sequestered was relatively small compared to the overall emissions. Some significant amounts of CO_2_ were sequestered in the simulation for a period of time, but this sink was eventually lost as the trees matured. Hence, tree planting as a geoengineering option appeared to provide only a temporary benefit. These results align with other research showing the challenges of not exceeding a 2 °C temperature rise by the end of the century.^42^

The results strongly suggest that the rise in global net radiative forcing and associated global temperature rise could be stabilized, at least for a period of time, by the deployment of geoengineering. Sea spray injection was clearly the preferred option in the simulations based on its cost and effectiveness. Sulfur injection appeared to be the next preferred option. Ocean fertilization was not called upon in any of the cases, so its cost‐effectiveness was clearly lower. A significant feature of the cost results was that geoengineering had a negligible impact on the overall global energy cost. The cost of deploying 25 m^3^ s^−1^ of sea spray injection is only $2.6 billion based on cost estimates in ref. 10. Similarly, the cost of deploying 2.5 million metric tons of sulfur injection as described in the GeoPen2‐CL case would involve a capital cost for the needed aircraft of roughly $2.63 billion and an annual cost for the deployment of $1.14 billion using the data from ref. 8. These amounts are small compared to overall global energy costs totaling trillions of dollars annually. Also of significance is that geoengineering still appeared to be cost effective even when a substantial penalty charge was applied. As shown in Table 5, the overall costs with a large penalty on geoengineering (the GeoPen‐CL and GeoPen2‐CL cases) were not that much higher than the baseline case (Base‐NL).

**Figure 18:**
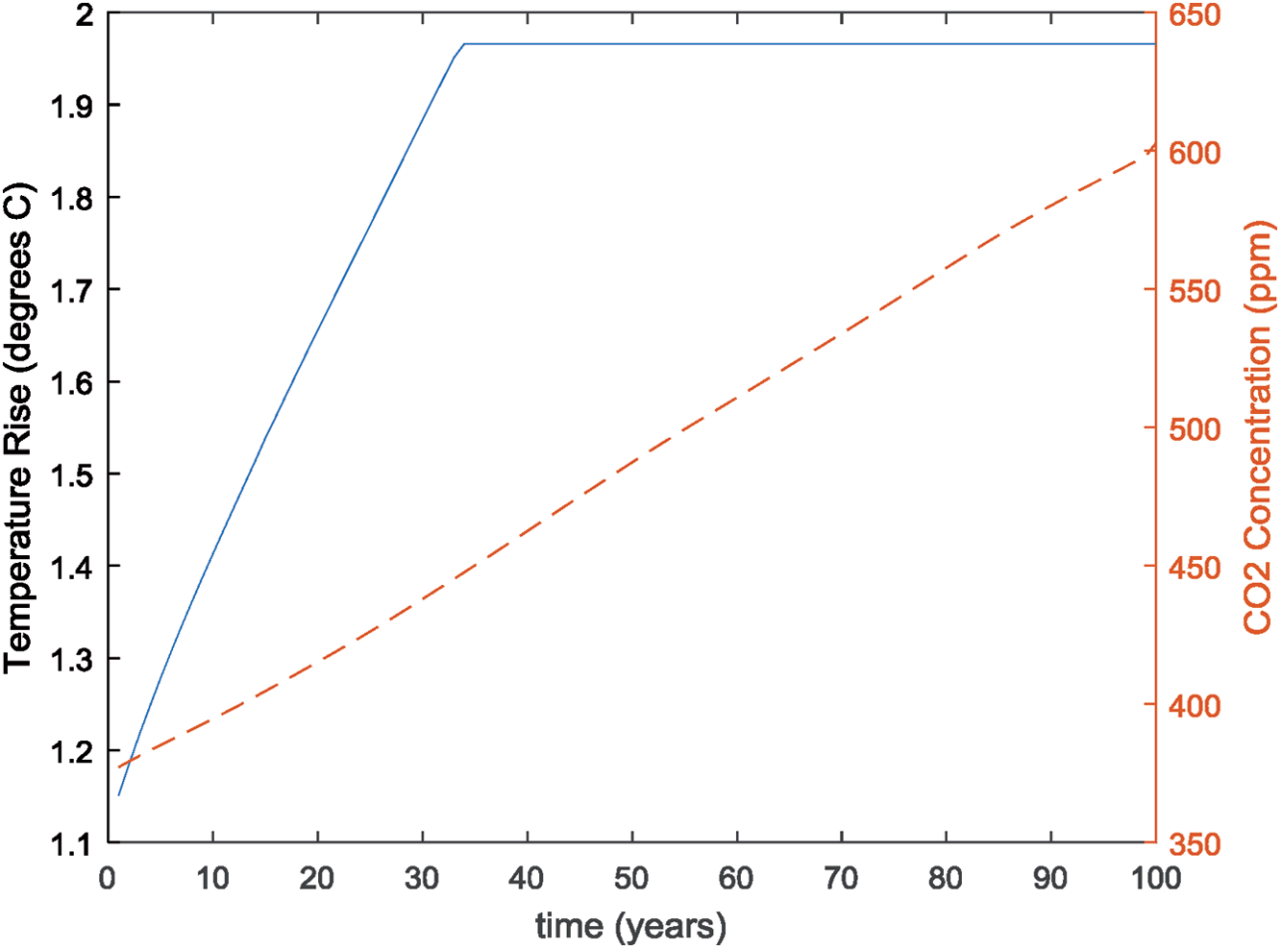
Temperature rise and atmospheric CO_2_ concentration in baseline case with geoengineering (Base‐CL).

**Figure 19:**
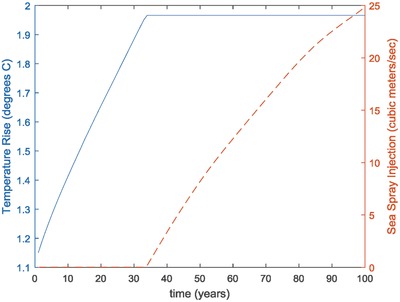
Temperature rise and sea spray injection in baseline case (Base‐CL).

These geoengineering results raise a number of important considerations. First of all, both sea spray and sulfur injection directly impact the earth's radiative forcing. They do not, however, do anything to alter the actual concentration of greenhouse gases in the atmosphere. Hence, even in the cases where geoengineering was deployed, actual emissions of GHG and their accumulation in the atmosphere would continue. This means that geoengineering would have to be continually deployed and deployed at an increasing rate in order to hold the net forcing and temperature rise constant. This is illustrated in **Figures** **18** and **19** which show the changes in temperature rise, atmospheric CO_2_ concentration, and sea spray deployment for the base case Base‐CL. A sudden cessation of geoengineering deployment would, therefore, result in a sudden increase in radiative forcing with a corresponding rise in global temperature. The implications of such an event are likely to be very severe.

Another serious and difficult to quantify concern is the potential negative impacts geoengineering could have on weather patterns and issues such as ocean acidification. Cases GeoPen and GeoPen2 attempted to address this issue by assigning high penalty costs to geoengineering as a means of accounting for at least some of these potential negative impacts. The thought was that these penalty costs might make geoengineering unattractive enough that other non‐GHG producing energy options might be favored by the model. As the simulation results showed, however, this was not the case. Geoengineering was still deployed even with the high penalty costs. Essentially, the specified climate limits could not be met without it. This is also borne out by the results of the NoGeo and NoGeo2 cases.

**Figure 20:**
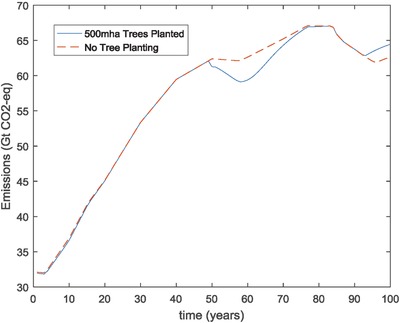
Comparison of annual emissions between baseline case (Base‐NL) and case with 500mha of trees planted in year 2050 (500mha‐CL).

The analysis also showed that significant amounts of tree planting on overall emissions and GHG emissions may be marginal. The amount of CO_2_ sequestered was relatively small compared to the overall emissions. The actual cumulative emissions in the 10mha‐CL and 500mha‐CL scenarios were 5600 Gt CO_2_‐eq and 5599 Gt CO_2_‐eq, respectively. These emissions were only slightly lower than the cumulative emissions in the base case of 5640 Gt CO_2_‐eq. Both sensitivity cases required nearly same amount of sea spray geoengineering as the base case Base‐CL in order to meet the climate target. Some significant reductions in emissions were realized for a period of time, but this sink for CO_2_ emissions was eventually lost as the trees matured. The benefit from the trees was, therefore, only temporary. This is because trees sequester CO_2_ as they grow and add more wood. Most of this growth and associated CO_2_ sequestration takes place early in the trees' life. As the trees mature and add less wood volume, the CO_2_ sequestration slows down and eventually stops once the trees have reached full maturity. This is illustrated in **Figure** **20** which compares the emissions in the 500mha‐CL case to the annual emissions for the base case Base‐NL. This is not to say that tree planting is not beneficial for a host of other environmental, social, and economic reasons. However, this analysis indicates that it cannot be relied upon as a permanent solution for curbing the impacts of increasing CO_2_ emissions.

Interestingly, the sensitivity cases with significant tree planting (10mha‐CL and 500mha‐CL) as well as the case with reduce acreage for tree planting and biofuel/biomass production (2bha‐CL) all showed relatively high overall costs. The reason for this was that these cases resulted in a reduction in the amount of biofuel that could be produced in the simulation. In the later years of the scenarios, the amount of oil available for use decreased as the proven reserves of this resource were used up. Biofuel was then used to meet the need for liquid fuels. When the amount of land available to produce biofuel in the simulation reached its maximum, very expensive coal‐to‐liquids technology had to be employed. Hence, any scenario that restricted the amount of biofuel that could be produced resulted in higher costs.

The electric vehicle sensitivity case (Elcar‐CL) provided some interesting insights. This scenario had the lowest cost on a net present value basis assuming a 4% discount rate. A key aspect of the scenario was that it significantly reduced the need for liquid fuel which meant that there was less depletion of oil reserves and, thus, less need for more costly biofuel and no need to rely on the expensive coal‐to‐liquids resource. Furthermore, this scenario had one of the lowest overall emissions of any of the scenarios. Hence, it required one of the lowest levels of geoengineering to meet the climate target. Only the case with a $100 per ton carbon tax required less geoengineering. Hence, this suggests that a major shift to electric vehicles could indeed provide significant climate benefits and reduce the overall global energy costs.

The analysis clearly demonstrated the sensitivity of the overall energy resource mix to the assumed discount rate. Lowering the discount rate from 4 to 1% significantly reduced the amount of nuclear and gas‐fired electricity generation with a corresponding increase in the deployment of wind. The use of coal also increased in the later years of the simulation compared to the base case with a 4% discount rate. However, coal‐to‐liquids did not need to be used at all in order to meet liquid energy demand in the later years. By contrast, a 10% discount rate resulted in a very large use of natural gas, especially for electricity generation, as well as large use of hydro, nuclear, and solar‐PV. Deployment of wind was considerably less than in the case with a 1% discount rate.

As noted earlier, the very high cost cases on a net present value basis were those that assumed limited use of nuclear power and a reduced amount of achievable energy efficiency. Throughout the various scenarios, energy efficiency and nuclear power were consistently favored. Furthermore, both were used to meet a substantial portion of the overall energy demand. In the case of nuclear power, this was even the case with a penalty charge applied. Hence, this implies that both of these energy resources could play a key role in optimally meeting future energy needs.

Employing a $100 per ton carbon tax yielded a significant reduction in GHG emissions and the lowest geoengineering requirement. The $50 per ton carbon tax had far less impact on meeting the climate limit. Interestingly, both carbon tax scenarios showed lower overall resource costs than the base case. In the base case, large amounts of oil and natural gas were used early in the simulation; however, their usage dropped off significantly after the year 2080 due to the depletion of the known reserves. As a result, more expensive biofuel and coal‐to‐liquids had to be used to make up the difference. In the carbon tax scenarios, lower amounts of these fossil fuels are used. Hence, the known reserves were not depleted nearly as much which resulted in less deployment of the more expensive biofuel and coal‐to‐liquids resources. As shown in Table 6, the carbon tax scenarios generated significant tax revenues. These tax revenues represent a transfer of income from businesses and consumers to governments. Therefore, there would likely be significant opposition to instituting such carbon taxes, especially since they would be an additional cost of doing business that is over and above the direct resource costs.

The two scenarios without geoengineering, NoGeo and NoGeo2, also showed surprising low overall resource costs. This was largely due to the much higher level of achievable energy efficiency assumed in those cases compared to the other scenarios. Energy efficiency had one of the lowest costs of any resource and was deployed to its fullest in both scenarios. These scenarios also resulted in large carbon tax revenues, as shown in Table 6, since they both assumed a $100 per ton carbon tax.

As with all models and simulations, there are important caveats regarding these results. First of all, the optimization is dependent on the assumed resource costs. Significant changes in cost assumptions can alter the results. Furthermore, as already demonstrated, the discount rate chosen will have a major impact on the results. In addition, the analysis is based on a single resource forecast. Significant changes in the energy forecast will change both the resource mix and the associated emissions which, in turn, will change the amount of geoengineering required. Finally, since the analysis only includes proven energy resources, any new technological breakthroughs could profoundly change how energy needs are met and the resulting impact to the climate. Despite these limitations, however, this analysis does show what may be required to meet a growing global demand for energy and meet necessary climate targets at the lowest cost.

## 6 Conclusions

Overall, the results of this analysis suggest several things. First of all, energy efficiency and nuclear power will likely play key roles in helping to achieve global greenhouse concentration and temperature goals while still meeting energy demands at a minimal cost. In the case of nuclear power, this is still the case even if penalty costs are included. Natural gas, biofuel, hydro, wind, and geothermal will also need to cover a major portion of future energy demand. Use of coal needs to be steadily ramped down over time. Carbon taxes can help to reduce fossil fuel use and associated GHG emissions, but need to be significant in order to make a difference and are likely to be highly controversial. Hence, an overall optimal strategy for the 21st century suggested by these results would, therefore, be one that would include the following features:

- An aggressive effort to maximize energy efficiency across all economic sectors and countries.
- Shifting 20% of the global vehicle fleet to electric cars.
- Promotion of hydro, nuclear, geothermal, and wind energy.
- Replace coal usage with natural gas wherever possible.
- Consideration of significant carbon taxes to discourage fossil fuel use.
- Extensive tree planting can be considered as an interim measure to help reduce emissions and allow time for new low‐carbon energy resources and technologies to be developed.
- Be prepared to deploy some level of geoengineering (either sea spray or sulfur injection) by the middle of the 21st century.

Finally and perhaps most importantly, the results of this analysis demonstrated the need for careful and coordinated policies across the world to support global temperature change limits. The analysis showed what an optimal mix of energy resources and geoengineering might look like to meet global energy demands subject to limits on GHG concentrations and associated global temperature change; however, since the analysis only went out to the year 2100, these results by no means guarantee permanent stabilization of the global temperature. That requires stabilization and potentially the reduction in actual GHG concentrations. Geoengineering options which only counteract the radiative forcing effects of greenhouse gases without reducing their actual concentration merely buy the world some time. Ultimately, policies and technologies will have to be put in place at some point in the future which stop GHG concentrations from rising if we are to prevent an uncontrolled rise in global temperature.

## Conflict of Interest

The authors declare no conflict of interest.

## Supporting information

Supplementary

## References

[1] Summary for Policymakers, IPCC, 2014: Climate Change 2014: Synthesis Report. Contribution of Working Groups I, II and III to the Fifth Assessment Report of the Intergovernmental Panel on Climate Change (Eds: Pachauri R. K., Meyer L. A.), IPCC, Geneva, Switzerland 2014, pp. 151.

[2] N. Stern , The Economics of Climate Change: The Stern Review, Cambridge University Press, Cambridge, UK 2007.

[3] Climatic Cataclysm: The Foreign Policy and National Security Implications of Climate Change (Ed: Campbell K.), Brookings Institution Press, Washington, D.C. 2008.

[4] W. Nordhaus , J. Econ. Perspect. 1993, 7, 11.

[5] “No to the Paris Accord”, National Review, December 14, 2015, http://www.nationalreview.com/article/428485/paris‐climate‐agreement‐just‐say‐no (accessed: August 2017).

[6] A. Abbot , Why Trump Can and Should Pull Out of Paris Climate Change Agreement, The Heritage Foundation, May 5, 2017 http://www.heritage.org/environment/commentary/why‐trump‐can‐and‐should‐pull‐out‐paris‐climate‐change‐agreement (accessed: August 2017).

[7] P. Crutzen , Clim. Change 2006, 77, 211.

[8] A. Robock , A. Marquardt , B. Kravitz , G. Stenchikov , Geophys. Res. Lett. 2009, 36, L19703.

[9] J. Latham , P. Rasch , C. Chen , L. Kettles , A. Gadian , A. Gettelman , H. Morrison , K. Bower , T. Choularton , Philos. Trans. R. Soc., A 2008, 366, 3969. 10.1098/rsta.2008.0137 18757272

[10] S. Salter , G. Sortino , J. Latham , Philos. Trans. R. Soc., A 2008, 366, 3989. 10.1098/rsta.2008.0136 18757273

[11] R. Lampitt , E. Achterberg , T. Anderson , J. Hughes , M. Inglesias‐Rodriguez , B. Kelly‐Gerreyn , M. Lucas , E. Popova , R. Sanders , J. Shepherd , D. Smythe‐Wright , A. Yool , Philos. Trans. R. Soc., A 2008, 366, 3919. 10.1098/rsta.2008.0139 18757282

[12] T. M. Lenton , N. E. Vaughan , Atmos. Chem. Phys. 2009, 9, 5539.

[13] V. Smetacek , C. Klaas , V. Strass , P. Assmy , M. Montresor , B. Cisewski , N. Savoye , A. Webb , F. d'Ovidio , J. Arrieta , U. Bathmann , R. Bellerby , G. Berg , P. Croot , S. Gonzalez , J. Henjes , G. Herndl , L. Hoffmann , H. Leach , M. Losch , M. Mills , C. Neill , I. Peeken , R. Rottgers , O. Sachs , E. Sauter , M. Schmidt , J. Schwarz , A. Terbruggen , D. Wolf‐Gladrow , Nature 2012, 487, 313. 22810695 10.1038/nature11229

[14] V. Smetacek , S. Naqvi , Philos. Trans. R. Soc., A 2008, 366, 3947. 10.1098/rsta.2008.0144 18757280

[15] G. Bala , Curr. Sci. 2009, 96, 41.

[16] A. Jones , J. Haywood , O. Boucher , Atmos. Sci. Lett. 2010, 10.1002/asl.291.

[17] H. D. Matthews , K. Caldeira , Proc. Natl. Acad. Sci. USA 2007, 104, 9949. 17548822 10.1073/pnas.0700419104 PMC1885819

[18] A. Robock , Bulletin of the Atomic Scientists, Taylor and Francis, Milton Park, UK 2008.

[19] A. Robock , L. Oman , G. Stenchikov , J. Geophys. Res. 2008, 113, D16101.

[20] J. Blackstock , D. Battisti , K. Caldeira , D. Eardley , J. Katz , D. Keith , A. Patrinos , D. Schrag , R. Socolow , S. Koonin , Climate Engineering Responses to Climate Emergencies, http://arxiv.org/pdf/0907.5140 (accessed: November 2009).

[21] K. Caldeira , G. Bala , L. Cao , Ann. Rev. Earth Planet. Sci. 2013, 41, 231.

[22] S. Schneider , Philos. Trans. R. Soc., A 2008, 366, 3843. 10.1098/rsta.2008.0145 18757279

[23] M. Weitzman , Some Basic Economics of Extreme Climate Change, https://scholar.harvard.edu/weitzman/publications/some‐basic‐economics‐climate‐change (February 2009).

[24] S. Barret , Environ. Resource Econ. 2008, 39, 45.

[25] National Academy of Sciences , Climate Intervention: Carbon Dioxide Removal and Reliable Sequestration, Committee on Geoengineering Climate: Technical Evaluation and Discussion of Impacts, Board on Atmospheric Sciences and Climate, Ocean Studies Board, Division of Earth and Life Sciences, National Academies Press, Washington, D.C. 2015.

[26] National Academy of Sciences , Climate Intervention: Reflecting Sunlight to Cool Earth, Committee on Geoengineering Climate: Technical Evaluation and Discussion of Impacts, Board on Atmospheric Sciences and Climate, Ocean Studies Board, Division of Earth and Life Sciences, National Academies Press, Washington, D.C. 2015.

[27] R. Loulou , U. Remne , A. Kanudia , A. Lehtila , G. Goldstein , Documentation for the TIMES Model, Part I, International Energy Agency, Energy Technology Systems Analysis Programme, April 2005.

[28] Energy Information Administration , Integrating Module of the National Energy Modeling System: Model Documentation 2014, July 2014.

[29] Joint Global Change Research Institute , GCAM v4.3 Documentation: GCAM Model Overview, http://jgcri.github.io/gcam‐doc/overview.html (accessed: April 2017).

[30] M. Labriet , R. Loulou , A. Kanudia , Les Cahiers du GERAD, G‐2008‐30, April 2008.

[31] J. G. Anasis , Dissertations and Theses, Paper 2620, Portland State University, 2015, http://pdxscholar.library.pdx.edu/open_access_etds/262010.15760/etd.2616.

[32] K. Anderson , G. Peters , Science 2016, 354, 182. 27738161 10.1126/science.aah4567

[33] O. Edenhofer , R. Pichs‐Madruga , Y. Sokona , S. Kadner , J. C. Minx , S. Brunner , S. Agrawala , G. Baiocchi , I. A. Bashmakov , G. Blanco , J. Broome , T. Bruckner , M. Bustamante , L. Clarke , M. Conte Grand , F. Creutzig , X. Cruz‐Núñez , S. Dhakal , N. K. Dubash , P. Eickemeier , E. Farahani , M. Fischedick , M. Fleurbaey , R. Gerlagh , L. Gómez‐Echeverri , S. Gupta , J. Harnisch , K. Jiang , F. Jotzo , S. Kartha , S. Klasen , C. Kolstad , V. Krey , H. Kunreuther , O. Lucon , O. Masera , Y. Mulugetta , R. B. Norgaard , A. Patt , N. H. Ravindranath , K. Riahi , J. Roy , A. Sagar , R. Schaeffer , S. Schlömer , K. C. Seto , K. Seyboth , R. Sims , P. Smith , E. Somanathan , R. Stavins , C. von Stechow , T. Sterner , T. Sugiyama , S. Suh , D. Ürge‐Vorsatz , K. Urama , A. Venables , D. G. Victor , E. Weber , D. Zhou , J. Zou , T. Zwickel , in Climate Change 2014: Mitigation of Climate Change. Contribution of Working Group III to the Fifth Assessment Report of the Intergovernmental Panel on Climate Change (Eds: Edenhofer O., Pichs‐Madruga R., Sokona Y., Farahani E., Kadner S., Seyboth K., Adler A., Baum I., Brunner S., Eickemeier P., Kriemann B., Savolainen J., Schlömer S., von Stechow C., Zwickel T., Minx J. C.), Cambridge University Press, Cambridge, NY, USA 2014.

[34] L. Clarke , K. Jiang , K. Akimoto , M. Babiker , G. Blanford , K. Fisher‐Vanden , J.‐C. Hourcade , V. Krey , E. Kriegler , A. Löschel , D. McCollum , S. Paltsev , S. Rose , P. R. Shukla , M. Tavoni , B. C. C. van der Zwaan , D. P. van Vuuren , in Climate Change 2014: Mitigation of Climate Change. Contribution of Working Group III to the Fifth Assessment Report of the Intergovernmental Panel on Climate Change (Eds: Edenhofer O., Pichs‐Madruga R., Sokona Y., Farahani E., Kadner S., Seyboth K., Adler A., Baum I., Brunner S., Eickemeier P., Kriemann B., Savolainen J., Schlömer S., von Stechow C., Zwickel T., Minx J.C.), Cambridge University Press, Cambridge, NY, USA 2014.

[35] G. Myhre , D. Shindell , F.‐M. Bréon , W. Collins , J. Fuglestvedt , J. Huang , D. Koch , J.‐F. Lamarque , D. Lee , B. Mendoza , T. Nakajima , A. Robock , G. Stephens , T. Takemura , H. Zhang , in Climate Change 2013: The Physical Science Basis. Contribution of Working Group I to the Fifth Assessment Report of the Intergovernmental Panel on Climate Change (Eds: Stocker T. F., Qin D., Plattner G.‐K., Tignor M., Allen S. K., Boschung J., Nauels A., Xia Y., Bex V., Midgley P. M.), Cambridge University Press, Cambridge, United Kingdom and New York, NY, USA **2013**, www.climatechange2013.org and www.ipcc.ch (accessed: October 2014).

[36] MacroTrends LIBOR Rates: 30 Year Historical Chart web site , http://www.macrotrends.net/1433/historical‐libor‐rates‐chart (accessed: December 2016).

[37] JPMorgan Chase Historical Prime Rate web site, https://www.jpmorganchase.com/corporate/About‐JPMC/historical‐prime‐rate.htm (accessed: December 2016).

[38] FAO Statistoical Yearbook **2013**: World Food and Agriculture, Food and Agriculture Organization of the United Nations, Rome 2013.

[39] R. Sims , R. Schaeffer , F. Creutzig , X. Cruz‐Núñez , M. D'Agosto , D. Dimitriu , M. J. Figueroa Meza , L. Fulton , S. Kobayashi , O. Lah , A. McKinnon , P. Newman , M. Ouyang , J. J. Schauer , D. Sperling , G. Tiwari , in *Climate Change 2014: Mitigation of Climate Change. Contribution of Working Group III to the Fifth Assessment Report of the Intergovernmental Panel on Climate Change* (Eds: Edenhofer O., Pichs‐Madruga R., Sokona Y., Farahani E., Kadner S., Seyboth K., Adler A., Baum I., Brunner S., Eickemeier P., Kriemann B., Savolainen J., Schlömer S., von Stechow C., Zwickel T., Minx J. C.), Cambridge University Press, Cambridge, NY, USA 2014.

[40] W. Krewitt , K. Nienhaus , C. Klebmann , C. Capone , E. Stricker , W. Graus , M. Hoogwijk , N. Supersberger , U. von Winterfeld , S. Samadi , Role and Potential of Renewable Energy and Energy Efficiency for Global Energy Supply, Project no. (FKZ) 3707 41 108, Report no. (UBA‐FB) 001323/E, 2009, report to the German Federal Environment Agency, http://www.umweltbundesamt.de/en/publikationen/role‐potential‐of‐renewable‐energy‐energy (accessed: September 2011).

[41] AR5 Scenario Database, https://secure.iiasa.ac.at/web‐apps/ene/AR5DB/dsd?Action=htmlpage&page=about (accessed: December 2014).

[42] J. Tollefson , Nature 2015, 527, 436. 26607526 10.1038/527436a

