# Supplementary material for: An Analysis of the Optimal Mix of Global Energy Resources and the Potential Need for Geoengineering Using the CEAGOM Model
Source: Glob Chall. 2017 Nov 13;1(8):1700040. doi: 10.1002/gch2.201700040 (PMC6607309; doi:10.1002/gch2.201700040)
Supplement: Supplementary file 1 — Supplementary [file GCH2-1-1700040-s001.pdf]

Copyright WILEY-VCH Verlag GmbH & Co. KGaA, 69469 Weinheim, Germany, 2017.

Global Challenges
Open Access

Supporting Information

for *Global Challenges,* DOI: 10.1002/gch2.201700040

An Analysis of the Optimal Mix of Global Energy Resources and the Potential Need for Geoengineering Using the CEAGOM Model

John G. Anasis, Mohammad Aslam Khan Khalil, George G. Lendaris, Christopher L. Butenhoff, and Randall Bluffstone*

Supporting Information

An Analysis of the Optimal Mix of Global Energy Resources and the Potential Need for Geoengineering Using the CEAGOM Model

John G. Anasis, Mohammad Aslam Khan Khalil, George G. Lendaris, Christopher L. Butenhoff,*

Randall Bluffstone

Table S.1. Energy and Geoengineering Resource Costs, Efficiencies, and Capacity Factors.

Resource	Cost or Efficiency Parameter	Value	Source
Oil	Variable Cost	$98/barrrel	Estimated from AEO2015 Brett price data
	Capital Cost	$33.8/barrel	EIA Website "How Much Does it Cost to Produce Crude Oil and Natural Gas", http://www.eia.gov/tools/faqs/faq.cfm?id=367&t=8
	Well to Tank (WTT) Efficiency	85%	Hekkert, M., F. Hendriks, A. Faaij, & M. Neelis, Natural Gas as an Alternative to Crude Oil in Automotive Fuel Chains Well-to-Wheel Analysis and Transition Strategy Development, Energy Policy, vol. 33, 2005, pp. 579-594.
	Engine Efficiency	33%	Sims,. R. et. al., 2014: Transport in: Climate Change 2014; Mitigation of Climate Change. Contribution of Working Group III to the Fifth Assessment Report of the Intergovernmental Panel on Climate Change, Cambridge University Press and also Technology Roadmap: Fuel Economy of Road Vehicles, International Energy Agency, 2012.
	Furnace Efficiency	85%	U.S. Department of Energy web site "Furnaces and Boilers", June 24, 2012, http://energy.gov/energysaver/articles/furnaces-and-boilers
	Well to Furnace Combustion Efficiency	72%	WTT efficiency x Furnace Efficiency
	Transport Efficiency	28%	WTT efficiency x Engine Efficiency

Natural Gas	Variable Cost	$5.62/Mcf	Independent Petroleum Association of America, United States Petroleum Statistics, 2011 Data, Published August 2012
	Capital Cost	$33.8/5618 cubic feet	EIA Website "How Much Does it Cost to Produce Crude Oil and Natural Gas", http://www.eia.gov/tools/faqs/faq.cfm?id=367&t=8
	Well to Tank (WTT) Efficiency	90%	Hekkert, M., F. Hendriks, A. Faaij, & M. Neelis, Natural Gas as an Alternative to Crude Oil in Automotive Fuel Chains Well-to-Wheel Analysis and Transition Strategy Development, Energy Policy, vol. 33, 2005, pp. 579-594.
	Furnace Efficiency	85%	U.S. Department of Energy web site "Furnaces and Boilers", June 24, 2012, http://energy.gov/energysaver/articles/furnaces-and-boilers
	Well to Furnace Combustion Efficiency	77%	WTT Efficiency x Furnace Efficiency
Natural Gas Fired Power Plant	Capital Cost	$718/kW	National Energy Technology Laboratory (NETL) Report Cost and Performance Baseline for Fossil Energy Plants, Volume 1 Bituminous Coal and Natural Gas to Electricity, DOE/NETL-2010/1397, Revision 2a, September 2013.
	Power Plant Efficiency	50%	National Energy Technology Laboratory (NETL) Report Cost and Performance Baseline for Fossil Energy Plants, Volume 1 Bituminous Coal and Natural Gas to Electricity, DOE/NETL-2010/1397, Revision 2a, September 2013, Page 5
	Well to Plant Efficiency	45%	WTT Efficiency x Generator Efficiency
Coal	Variable Cost	$39.9/short ton ($43.9/metric ton)	EIA Annual Energy Outlook 2014 Early Release Overview, December 16, 2013
	Mine Capital Cost	$550 million Au/21,800 metric tons	Shafiee, S., M. Nehring, and E. Topal, Estimating Average Total Cost of Open Pit Coal Mines in Australia, Australian Mining Technology Conference, Oct. 27-28, 2009, pp. 134-145
	Mine to Tank (MTT) Efficiency	93%	Odeh, N., & T. Cockerill, Life Cycle GHG Assessment of Fossil Fuel Power Plants with Carbon Capture and Storage, Energy Policy, vol. 36, 2008, pp. 367-380.

	Furnace Efficiency	85%	U.S. Department of Energy web site "Furnaces and Boilers", June 24, 2012, http://energy.gov/energysaver/articles/furnaces-and-boilers
	Mine to Furnace Combustion Efficiency	79%	MTT Efficiency x Furnace Efficiency
Coal Fired Power Plant	Plant Capital Cost	$2.02 million/MW	National Energy Technology Laboratory (NETL) Report Cost and Performance Baseline for Fossil Energy Plants, Volume 1 Bituminous Coal and Natural Gas to Electricity, DOE/NETL-2010/1397, Revision 2a, September 2013.
	Total Capital Cost	$24,600/metric ton	Power Plant Plus Mine Capital Costs
	Decommissioning Cost	$1 million/MW	Electric Power Research Institute, Decommissioning Handbook for Coal-Fired Power Plants, document 1011220, 2004
	Generator Efficiency	39%	National Energy Technology Laboratory (NETL) Report Cost and Performance Baseline for Fossil Energy Plants, Volume 1 Bituminous Coal and Natural Gas to Electricity, DOE/NETL-2010/1397, Revision 2a, September 2013.
	Mine to Plant Efficiency	36%	MTT Efficiency x Generator Efficiency
Coal-to-Liquids	Non-Fuel Operating Cost	$12/barrel	Bartis, J., F. Camm, & D. Ortiz, Producing Liquid Fuels from Coal: Prospects and Policy Issues, Rand Corporation, 2008.
	Variable Cost	$60.7/metric ton	Non-Fuel Operating Cost Plus Coal Variable Cost
	Plant Capital Cost	$125,000/barrel per day	Bartis, J., F. Camm, & D. Ortiz, Producing Liquid Fuels from Coal: Prospects and Policy Issues, Rand Corporation, 2008.
	Total Capital Cost	$199,000/metric ton	Plant Plus Mine Capital Costs
	Mine to Tank (MTT) Efficiency	93%	Odeh, N., & T. Cockerill, Life Cycle GHG Assessment of Fossil Fuel Power Plants with Carbon Capture and Storage, Energy Policy, vol. 36, 2008, pp. 367-380.
	Engine Efficiency	33%	Sims,. R. et. al., 2014: Transport in: Climate Change 2014; Mitigation of Climate Change. Contribution of Working Group III to the Fifth Assessment Report of the Intergovernmental Panel on Climate Change, Cambridge University Press and also Technology Roadmap: Fuel Economy of

			Road Vehicles, International Energy Agency, 2012.
	Mine to Engine Efficiency	16%	MTT Efficiency x Engine Efficiency x CTL Plant Efficiency
Nuclear	Variable Cost	$10/MW-h	Roques, F., W. Nuttall, D. Newbery, R. de Neufville, & S. Connors, Nuclear Power: A Hedge Against Uncertain Gas and Carbon Prices?, The Energy Journal, Vol. 27. No. 4, 2006.
	Waste Disposal Cost	$20/MW-h	Power Engineering, Dec. 20, 2013, web site: http://www.power-eng.com/articles/2013/12/nuclear-waste-storage-could-add-more-than-350bn-to-cost-of-nuclear.html
	Capital Cost	$2300/kW-h	Congressional Budget Office Cost Estimate of S. 14 Energy Policy Act of 2003, May 7, 2003
	Decommissioning Cost	$350 million/GW	Nuclear Regulatory Commission web site: http://www.nrc.gov/reading-rm/doc-collections/fact-sheets/decommissioning.html
	Capacity Factor	91%	Nuclear Energy Institute web site: http://www.nei.org/Knowledge-Center/Nuclear-Statistics/US-Nuclear-Power-Plants
Hydro	Capital Cost	$2950/kW	Updated Capital Cost Estimates for Utility Scale Electricity Generating Plants, Energy Information Administration, US Dept. of Energy, April, 2013
	Decommissioning Cost	$885/kW (30% of original construction cost)	International Water Power and Dam Construction Magazine web site: http://www.waterpowermagazine.com/features/featuredecommissioning-dams-costs-and-trends/
	Capacity Factor	40%	Energy Information Administration, Electric Power Annual 2009, DOE/EIA-0348(2009), Released Nov. 23, 2010, Revised Jan. 2011 & April 2011, Table 5.2
Wind	Capital Cost	$2250/kW	Updated Capital Cost Estimates for Utility Scale Electricity Generating Plants, Energy Information Administration, US Dept. of Energy, April, 2013
	Capacity Factor	30%	Crawford, R., Life Cycle Energy and Greenhouse Emissions Analysis of Wind Turbines and the Effect of Size on Energy Yield, Renewable and Sustainable Energy Reviews, vol. 13, 2009, pp. 2653-2661

Geothermal	Capital Cost	$4460/kW	Updated Capital Cost Estimates for Utility Scale Electricity Generating Plants, Energy Information Administration, US Dept. of Energy, April, 2013
	Capacity Factor	81%	Fredleifsson, I., Geothermal Energy for the Benefit of the People, Renewable and Sustainable Energy Reviews, vol. 5, 2001, pp. 299-313
Solar-PV	Capital Cost	$4210/kW	Updated Capital Cost Estimates for Utility Scale Electricity Generating Plants, Energy Information Administration, US Dept. of Energy, April, 2013
	Capacity Factor	20%	U.S. Department of Energy, Sunshot Vision Study, February, 2012
Solar-Thermal	Capital Cost	$5110/kW	Updated Capital Cost Estimates for Utility Scale Electricity Generating Plants, Energy Information Administration, US Dept. of Energy, April, 2013
	Capacity Factor	56%	National Renewable Energy Laboratory, Assessment of Parabolic Trough and Power Tower Solar Technology Cost and Performance Forecasts, NREL/SR-550-34440, October, 2003
Biofuel	Variable Cost	$2.49/gallon	Radich, A., Biodiesel Performance, Costs, and Use, Energy Information Administration web site:http://www.eia.gov/oiaf/analysispaper/biodiesel/
	Capital Cost	0.4 euro/liter	Charles, C., I. Gerasimchuk, R. Bridle, E. Asmelash, & T. Laan, Biofuels - At What Cost?, A Review of Costs and Benefits of EU Biofuel Policies, International Institute for Sustainable Development, Global Subsidies Initiative, Research Report Technical Annex, April, 2013
	Farm to Tank (FTT) Efficiency	79%	Hill, J., E. Nelson, D. Tilman, S. Polasky, & D. Tiffany, Environmental, Economic, and Energetic Costs and Benefits of Biodiesel and Ethanol Biofuels, Proceedings of the National Academy of Sciences, vol. 103, no. 30, July 25, 2006, pp. 11206-11210
	Engine Efficiency	29%	Radich, A., Biodiesel Performance, Costs, and Use, Energy Information Administration web site:http://www.eia.gov/oiaf/analysispaper/biodiesel/
	Farm to Engine Efficiency	23%	FTT Efficiency x Engine Efficiency

Biomass	Variable Cost	$5.26/kW-year	Updated Capital Cost Estimates for Utility Scale Electricity Generating Plants, Energy Information Administration, US Dept. of Energy, April, 2013
	Capital Cost	$4220/kW	Updated Capital Cost Estimates for Utility Scale Electricity Generating Plants, Energy Information Administration, US Dept. of Energy, April, 2013
	Capacity Factor	83%	Tidball, R, J. Bluestein, N. Rodriguez, & S. Knoke, Cost and Performance Assumptions for Modeling Electricity Generation Technologies, National Renewable Energy Laboratory, Report NREL/SR-6A20-48595, November, 2010.
Energy Efficiency	Capital Cost	1.57E-5 *GDP ($trillion/EJ)	Krewitt, W., K. Nienhaus, C. Klebmann, C. Capone, E. Stricker, W. Graus, M. Hoogwijk, N. Supersberger, U. von Winterfeld, & S. Samadi, Role and Potential of Renewable Energy and Energy Efficiency For Global Energy Supply, German Federal Ministry of the Environment, Nature Conservation, and Nuclear Safety, Project No. (FKZ) 3703 41 108, Report No. (UBA-FB) 001323/E, December, 2009
Sulfur Injection	Variable Cost	$455 million/metric ton	Robock, A., A. Marquardt, B. Kravitz, & G. Stenchikov, Benefits, Risks, and Costs of Stratospheric Geoengineering, Geophysical Research Letters, vol. 36, L19703, 2009
	Capital Cost	$1.05 billion/metric ton	Robock, A., A. Marquardt, B. Kravitz, & G. Stenchikov, Benefits, Risks, and Costs of Stratospheric Geoengineering, Geophysical Research Letters, vol. 36, L19703, 2009
Iron Seeding	Variable Cost	$6750/metric ton	Wikipedia article on Iron Fertilization, web site: http://en.wikipedia.org/wiki/Iron_fertilization#cite_note-46
	Capital Cost	$58 million/100,000 metric tons	Wiki Answers web site "How Much Does it Cost to Build an Oil Tanker", http://wiki.answers.com/Q/How_much_does_it_cost_to_build_an_oil_tanker
Tree Planting	Capital Cost	$1500/ha	van Kooten, G., S. Shaikh, & P. Suchanek, Mitigating Climate Change by Planting Trees: The Transaction Costs Trap, Land Economics, Vol. 78, No. 4, November, 2002, pp. 559-573

Sea Spray Injection	Capital Cost	2 million pound sterling/ship	Salter, S., G. Sortino, & J. Latham, Sea-Going Hardware for the Cloud Albedo Method of Reversing Global Warming, Philosophical Transactions of the Royal Society A, vol. 366, 2008, pp. 3989-4006.
